# Beyond Sensitive and Selective Electrochemical Biosensors: Towards Continuous, Real-Time, Antibiofouling and Calibration-Free Devices

**DOI:** 10.3390/s20123376

**Published:** 2020-06-16

**Authors:** Susana Campuzano, María Pedrero, Maria Gamella, Verónica Serafín, Paloma Yáñez-Sedeño, José Manuel Pingarrón

**Affiliations:** Departamento de Química Analítica, Facultad de CC. Químicas, Universidad Complutense de Madrid, E-28040 Madrid, Spain; mpedrero@quim.ucm.es (M.P.); mariagam@quim.ucm.es (M.G.); veronicaserafin@quim.ucm.es (V.S.); yseo@quim.ucm.es (P.Y.-S.)

**Keywords:** electrochemical biosensors, real-time, continuous operation, reagentless, reusable, calibration-free, antibiofouling

## Abstract

Nowadays, electrochemical biosensors are reliable analytical tools to determine a broad range of molecular analytes because of their simplicity, affordable cost, and compatibility with multiplexed and point-of-care strategies. There is an increasing demand to improve their sensitivity and selectivity, but also to provide electrochemical biosensors with important attributes such as near real-time and continuous monitoring in complex or denaturing media, or in vivo with minimal intervention to make them even more attractive and suitable for getting into the real world. Modification of biosensors surfaces with antibiofouling reagents, smart coupling with nanomaterials, and the advances experienced by folded-based biosensors have endowed bioelectroanalytical platforms with one or more of such attributes. With this background in mind, this review aims to give an updated and general overview of these technologies as well as to discuss the remarkable achievements arising from the development of electrochemical biosensors free of reagents, washing, or calibration steps, and/or with antifouling properties and the ability to perform continuous, real-time, and even in vivo operation in nearly autonomous way. The challenges to be faced and the next features that these devices may offer to continue impacting in fields closely related with essential aspects of people’s safety and health are also commented upon.

## 1. Introduction

The availability of technologies for tracking the levels of specific molecules in real time in food production lines or in the living body would revolutionize various applications involved in aspects of people’s life safety and physical health, such as clinical diagnosis, food analysis, or environment monitoring [1,2]. 

However, other than for glucose, point-of-care molecular testing (POCT) is largely restricted to lateral-flow dipstick tests, a technology that is often hard to adapt to multiplexed detections and quantitative measurements [3]. Even so, this technology is making great strides and some methods do allow analyte quantification [4,5].

Motivated by overcoming these limitations and with the aim of improving the adaptation to the real world, electrochemical biosensors, i.e., those incorporating a biological molecule (enzyme, antibody, oligonucleotide, peptide, etc.) as recognition element, pursue features other than high sensitivity and selectivity. These aimed characteristics imply near real-time and continuous monitoring “at home” of molecular targets directly in complex or denaturing media, even under flowing conditions after minimal intervention. Successful meeting of these challenges requires the development of electrochemical devices with antibiofouling properties that are reagentless, single-step, no-wash, and calibration-free.

The great advances in recent years regarding the modification of electrode surfaces with nanomaterials and antibiofouling reagents as well as the irruption of folding-based biosensors have led to the development of electroanalytical bioplataforms filling many of these attributes. They allow the continuous, real-time, in situ measurement of specific molecules directly in flowing complex food samples [6], biological fluids [3,7,8,9], or even in the bodies of awake [10,11] and free moving individuals. 

With this background in mind, this review aims to give an updated overview of the main technologies and recent advances involving electrochemical biosensors free of reagents, washing, or calibration steps, and/or with antifouling properties useful for performing the determination of relevant molecular targets in untreated complex samples or after minimal pre-treatment both in vitro and in vivo. As far as we know, this is the first review that critically and jointly discusses the achievement of these outstanding attributes in electrochemical biosensing. 

## 2. Continuous, Real-Time Electrochemical Biosensors: Towards Antibiofouling, Reagentless, No-Wash, Single-Step, Reusable, and Calibration-Free Devices

Electrochemical biosensors exhibit distinct advantages compared to other biosensors, such as a lack of the high complexity of the sensor setup and the high cost. They are also robust, easy to miniaturize or multiplex, involve low-cost and portable instrumentation, and provide low detection limits even when small sample volumes are available. Furthermore, they can be used to analyze turbid fluids with optically absorbing or fluorescing compounds. 

Electrochemical biosensors have evolved as attractive tools to perform single or multiplexed determinations of molecular targets in a simple, affordable, and decentralized way. Although they are constantly seeking higher sensitivity and specificity, in addition, they search other particularly challenging attributes essential to make the reality of their marketing and implementation in the real world to come true. 

Currently, scientists are more and more aware that the single-handed pursuit of the sensitivity and accuracy cannot meet the demands of many in situ or POCT circumstances, especially in the fields of clinical diagnosis, food analysis, and environment monitoring. Increasing attention is focused on simplifying their operation and reducing detection time by developing no-wash electrochemical sensors, which make them more suitable for application in the in situ and POCT circumstances [1].

In addition, the ability to monitor specific molecules in real-time would greatly enhance the understanding of diseases as well as their early detection, monitoring, and treatment, thus helping to achieve the promise of personalized medicine. Moreover, triggering of timely countermeasure actions in the food safety and environmental fields would be expected. However, to achieve these goals, sensors must: (i) provide relevant selectivity, precision, and sensitivity; (ii) operate continuously with no sample preparation, batch processing (such as washing steps), or addition of exogenous reagents; and (iii) be insensitive to biofouling such as the detrimental accumulation of proteins and blood cells on the sensor surface [12].

Furthermore, the development of quantitative single-step and calibration-free biosensors is particularly relevant for sensors deployed in vivo to minimize the variability of their fabrication and baseline drift. The willingness to control test conditions and perform calibrations is not only inconvenient but impossible in these operating conditions [13].

The electrode surface modification with antibiofouling reagents, the rapid growth of nanoscience and nanotechnology, and the great advances experienced by folded-based biosensors and in the detection strategies have imparted upon electrochemical biosensors these highly pursued attributes. The remarkable characteristics achieved by electrochemical biosensors beyond sensitivity and selectivity are discussed below based on representative examples selected from recent literature and summarized in Table 1 to provide an overall picture. They are grouped in the following sections according to the more remarkable attribute exhibited. However, it is worth noting that many of these biosensors have additional features with great practical relevance. The important advances provided by wearable devices for real-time electrochemical biosensing have been extensively reviewed in the last few years [14,15,16,17,18,19] and are not discussed in this article. 

### 2.1. Electrochemical Biosensors with Antibiofouling Properties

The impressive opportunities and capabilities that electrochemical biosensors offer for the monitoring of a wide variety of molecules in situ in complex or biological fluids over a prolonged period of time are limited by the gradual passivation of the (bio)sensing surface due to the nonspecific accumulation of macromolecules present in such matrices. These biofouling issues reduce the direct contact of the target analyte with the electrode surface, hampering the electron transfer, and may severely affect the sensitivity, reproducibility, stability, and overall reliability of the resulting (bio)sensors [29]. Therefore, the development of biosensors with antibiofouling properties able to keep their performance after direct/prolonged incubation in complex and protein-rich media has encouraged the utmost interest. In order to do this, the sensing interfaces are modified with several kinds of antifouling materials, such as poly(ethylene glycol) (PEG) and oligo(ethyleneglycol) (OEG). Although these are considered as the “gold standard” materials, they show various defects such as their oxidative damage, poor water-solubility, and low protein resistance at high temperature. Peptides have come up as possible alternative antifouling materials in electrochemical affinity biosensors [30,31,32], providing additional advantages of biocompatibility, tunable and simple structure and synthesis [24]. 

Among all the strategies currently available to develop antibiofouling surfaces, the modification of electrode substrates with different biomaterials, including monolayers, transient polymeric coatings, or multifunctional peptides, is particularly attractive and promising. These strategies have been recently reviewed [29], and, therefore, this section discusses only remarkable features of a limited number of selected methods. 

A wide variety of monolayers that exhibit biofouling properties have been reported in the last decade [12,20,21,33,34,35,36,37,38,39,40,41,42,43,44,45,46,47,48,49,50,51].

In this context, binary monolayers involving thiolated nucleic acid capture probes (SHCP) and MCH self-assembled onto gold electrodes display unspecific background contributions, due to incomplete backfilling, and irreproducibility, which is attributed to the presence of surface defects and heterogeneity in the distribution of SHCP strands. This unspecific background negatively affects the performance of binary monolayers in complex biofluids and the storage stability of the resulting modified surfaces due to the displacement of SHCP by MCH [52]. 

**Figure 1:**
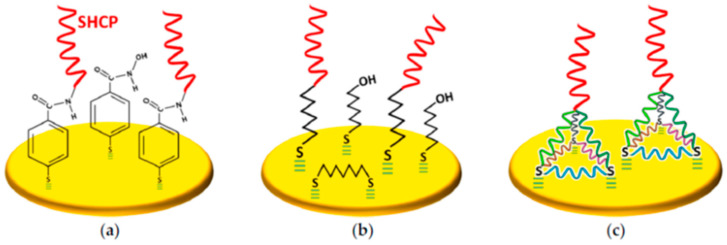
Schematic illustration of three different antifouling thiolated monolayers: (**a**) layers prepared by attaching NH_2_-CP to p-MBA SAM-modified electrodes, (**b**) ternary SHCP/HDT+MCH layers, and (**c**) layers prepared with TDNs. Reprinted from [29] with permission.

Recent research has shown that a judicious design of thiolated surface chemistry involving binary or ternary mixed monolayers, prepared by co-assembling or sequential assembly (noted by “/” or “+”, respectively, in their nomenclature) of the components, or the use of tetrahedral DNA nanostructures (TDNs), has led to electrochemical nucleic acid biosensors with substantially better antibiofouling properties and analytical characteristics as compared to conventional SHCP+MCH binary monolayers. This is the case, for example, of binary layers prepared by bringing SHCP into p-aminothiophenol (p-ATP) monolayers previously subjected to potential cycling at acidic pH [49], or layers prepared by attaching amino-functionalized capture probes (NH_2_-CP) to p-mercaptobenzoic acid (p-MBA) SAM-modified electrodes (Figure 1a) [48]. 

Pioneering work by Dharuman’s group described simultaneous control of probe orientation and surface passivation by ternary mixed monolayers prepared by sequential immobilization of thiolated ss-DNA probes, MCH, and mercaptopropionic acid (MPA) as diluents, also achieving higher hybridization and discrimination efficiencies due to the distance among anchored ssDNA probes. Moreover, MPA was demonstrated to be more effective than MCH in reducing unspecific adsorptions, due to the generated hydrogen bonds between MPA and MCH, and by placing the DNA strands perpendicularly to the electrode surface [53]. 

Wang’s group were pioneers in preparing ternary DNA SAM-interfaces by co-immobilizing a short (cyclic or linear) dithiol with the SHCP, followed by assembling MCH. The ternary interfaces provided significantly larger signal-to-blank ratios (~100-fold improvement) than the common binary SHCP+MCH SAMs [20,21,36,54]. Results obtained with different lineal dithiols (dithiothreitol, DTT, 1,3-propanedithiol, PDT, 1,6-hexanedithiol, HDT, and 1,9-nonanedithiol, NDT) showed that the SAMs formed with PDT and HDT exhibited better analytical performance due to the preferential lying-down orientation adopted by these linear dithiols. The dithiol lying down configuration minimized nonspecific adsorptions while maintaining SHCP favorable orientation and good permeability of small signaling molecules when compared to the compact surface coverage obtained with the ternary DTT SAM, which resulted in higher signals. The smaller signals observed at interfaces modified with NDT were attributed to the lower amount of attached SHCP [20]. 

The SHCP/HDT+MCH layers (Figure 1b), assembled onto photolithography-prepared Au electrode arrays [20] or gold screen-printed electrodes [21], exhibited better storage stability than the binary SHCP+MCH layers, and excellent resistance to fouling after 24 h incubation in undiluted human serum and urine. The as-prepared biosensors allowed direct measurement of target nucleic acid concentrations at pM levels in these raw liquid biopsies. 

TDNs are assembled on a gold surface through the reproducible immobilization of four especially designed ss-DNA strands, which constitute the six edges of a DNA tetrahedron. Thiol linkers are used at the ends of three component strands, and a linear sequence at the fourth vertex at the top of the bound tetrahedron is left pendant to anchor bio-probes (Figure 1c) [55,56]. This simple, rapid, and high yield one-step process leads to rigid, stable, and reproducible scaffolds adequate for anchoring recognition probes in an upright orientation, spatially segregated, and far away from electrode surfaces, in a solution-phase-like environment ensuring optimal hybridization without requiring a subsequent backfilling step [57]. These tetrahedral DNA nanostructured scaffolds exhibited higher stability and affinity and are less-susceptible to non-specific adsorptions than those fabricated with single point-tethered oligonucleotides [58].

Cell membrane-mimicking phosphatidylcholine (PC)-terminated monolayers are also an attractive option to prepare antibiofouling electrochemical biosensors. PC head-groups mimic the fouling resistance of eukaryotic cellular membranes by strongly binding water to produce a hydration layer that forms a barrier against protein or cell non-specific adsorption. These PC-terminated SAMs can be relatively short, thus supporting rapid electron transfer [12].

**Figure 2:**
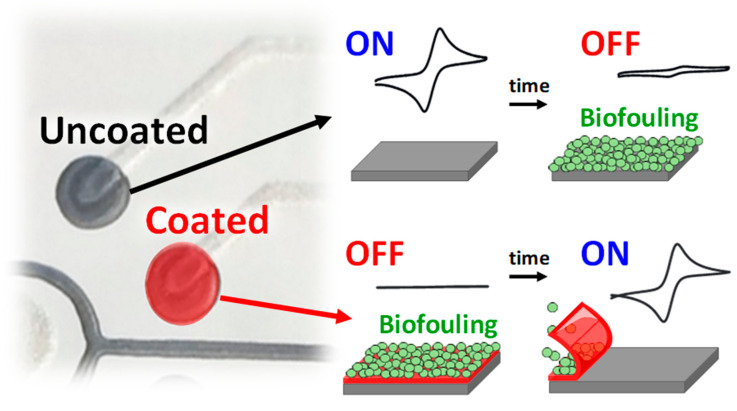
Schematic display of the methodology involving the use of biocompatible pH-sensitive transient polymer coatings to impart electrochemical biosensor antibiofouling properties. Figure drawn based on [22].

The use of biocompatible pH-sensitive transient polymer coatings has been recently exploited by Wang´s group to develop electrochemical biosensors that exhibit antibiofouling properties and good performance after prolonged incubation in complex biological fluids or media with denaturing pH values [22,23]. The method is based on the use of commercial biocompatible polymers sensitive to pH and of controlled dissolution, which are deposited on the (bio)sensor surface temporarily protecting it from undesirable adsorption processes during its prolonged incubation in the biological fluid of interest. The dissolution of these temporary methacrylate-based coatings can be controlled by varying the density and/or thickness of the deposited polymer layer, which allows leaving the "intact" (bio)sensing surface exposed only at the desired moment (Figure 2).

This strategy was applied to a four working electrodes-multisensing platform coated with different layers of Eudragit L100 polymer (which dissolves at pH ≥ 6). The sensing platform exhibited excellent operational characteristics in terms of reproducibility and controlled coating dissolution/tunable sequential actuation (0, 2, 4, or 6 h) of the individual electrodes. Monitoring was carried out by cyclic voltammetry with the [Fe(CN)_6_]^4−/3−^ redox system. The practical usefulness of this antibiofouling strategy was demonstrated with glucose enzyme biosensors, allowing the sequential enzymatic actuation every 2 h (0, 2, 4, or 6 h) and the direct glucose monitoring in untreated blood and saliva samples over prolonged periods (2 h) without compromising the sensitivity of the biosensors [22]. The excellent antifouling properties imparted by the temporary coatings allowed coated biosensors to maintain 100% of the initial response after 2 h of incubation in these complex biological fluids in comparison with the 65–70% lost observed for the unmodified biosensors. 

The unique advantages imparted by pH-responsive protective coatings were also exploited to ensure enzyme activity in media of denaturing pH values to develop edible electrochemical biosensors (based on carbon-paste prepared from olive oil, activated charcoal, and glucose oxidase) with remarkable prolonged resistance to extreme acidic conditions for glucose sensing in gastrointestinal fluids [23]. The active surface of the edible biosensors was modified with commercial polymers such as Eudragit E PO or L-100, which are dissolved at pHs ≤ 5.0 or ≥ 6.0, respectively, for dissolution in gastric fluid (pH between 1.5–5) or intestinal fluid (pH 6.5). The combination of edible olive oil-carbon pastes and transitory coatings preserved the catalytic activity of biomolecules in strongly acidic gastrointestinal fluids and protected the active surface of the biosensor from nonspecific adsorptions, allowing the dissolution/tuning activation of the biosensor selectively in gastric or intestinal fluids at previously fixed times.

**Figure 3:**
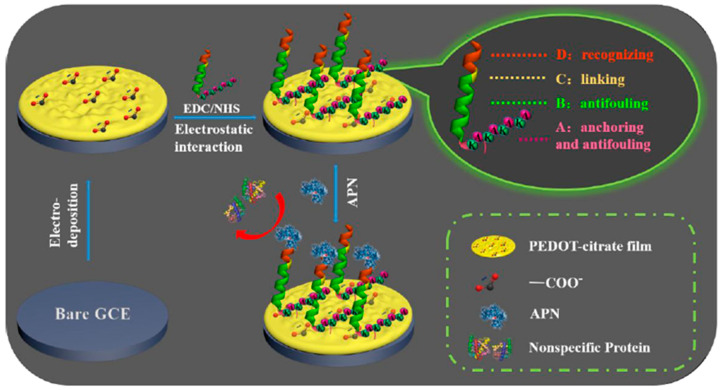
Electrochemical biosensor with antibiofouling properties for the determination of APN and HepG2 cells involving the immobilization of a multifunctional peptide onto a GCE modified with a PEDOT-citrate film. Reprinted from [24] with permission.

Some peptide sequences have shown good antifouling performances. However, complicated chemical reactions or self-assembling on metal surfaces like Au are usually employed for their immobilization at different surfaces, and they need additional reagents to block the peptide-modified interface [24]. To overcome these disadvantages, Song et al. [24] have recently proposed the preparation of an antifouling biosensor for the determination of both aminopeptidase N (APN) and human hepatocellular carcinoma cells (HepG2 cells). The preparation of the biosensor involved a GCE modified with electrodeposited poly (3,4-ethylenedioxythiophene) (PEDOT)-citrate film and the use of a multifunctional peptide with anchoring, antifouling, and recognizing abilities (Figure 3). In the designed three-in-one peptide, one end is a unique anchoring part rich in amine groups to allow its covalent immobilization using carbodiimide/succinimide (EDC/NHS) chemistry on GCE/PEDOT-citrate. The other end is the recognized part for target molecules, while the middle and the anchoring sides are designed to be antifouling. The as-devised biosensor showed, using DPV in the presence of [Fe(CN)_6_]^4−/3−^, antifouling properties after incubation in different charged protein solutions and human serum, as well as high sensitivity for the determination of the target analytes with detection limits of 0.4 ng mL^−1^ and 20 cells mL^−1^ for APN and HepG2 cells, respectively, in human urine.

It is also important to note at this point that the electrochemical switch-based biosensors have demonstrated to be less prone to fouling because of their transduction mechanism (this issue is discussed in more detail in the next section) [59,60].

### 2.2. Reagentless, No-Wash, Single-Step, Near Real-Time, and Reusable Electrochemical Biosensors

In the field of reagentless, real-time, and continuous monitoring, folding-based electrochemical biosensors have a fundamental role [59,60,61,62]. Recently, we have comprehensively reviewed the main features of this particular type of electrochemical biosensors [60]. Therefore, this section deals only with the relevant aspects within the context of this review article and addresses some methods that have emerged very recently.

Switch-based electrochemical biosensors use biomolecular switches, DNAs, aptamers, or peptides that reversibly change between at least two conformations in response to the specific binding of a wide range of molecular targets. The switches are modified with a linker for their immobilization on an interrogating electrode and at least one redox-active reporter [59,60]. The enzyme-free conformation-linked signal transduction mechanism relies on the target binding induction of a change in the conformation of the probe, which alters the efficiency with which the redox reporter transfers electrons to the electrode. This produces an easily measured signal, using common electrochemical techniques, which makes these biosensors rapid (often reaching effective equilibrium in seconds), drastically simple, and quite insensitive to nonspecific adsorption and response variability [63]. 

They can be classified as electrochemical DNA (E-DNA) [6,8,25] aptamer (E-AB) [2,3,7,9,10,26], and peptide (E-PB) [27] biosensors, and electrochemical biosensors for ion determination (E-ION) [28]. They have targeted the single or simultaneous determination [10,25,64,65,66] of a great number of significant analytes (DNAs, polymerase chain reaction amplification products, proteins, hormones, autoantibodies, drugs, toxins, adulterants, explosives, ions, and other biologically relevant molecules), and provide LODs as low as aM-fM for target DNAs [8,67,68], and pM for autoantibodies [69] and proteins [70], with compliance with threshold values and current regulations.

**Figure 4:**
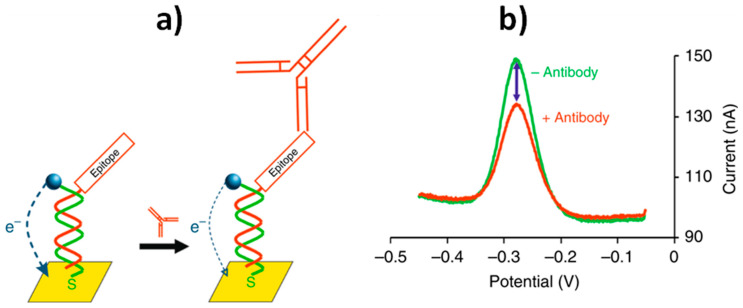
E-DNA sensor developed for the multiplexed determination of HIV-diagnostic antibodies using a nucleic acid “scaffold” anchored on one end to an electrode and presenting both a redox reporter and a specific epitope on the other. When the targeted antibody is not present, the DNA scaffold efficiently transfers electrons to the gold electrode, the electron transfer being reduced due to steric hindrance upon antibody binding (**a**); the square wave voltammetric signals obtained in the absence and in the presence of the targeted antibody (**b**). Reprinted and adapted from [25] with permission.

An illustrative example is shown in Figure 4, where the scheme of a recently reported E-DNA sensor for the multidetermination of three HIV-diagnostic antibodies in human serum [25] is displayed. The comparison of the biosensor performance with those of gold standard serological techniques shows that this strategy merged the quantitation and multiplexing of ELISAs with the convenience and speed of dipsticks.

**Figure 5:**
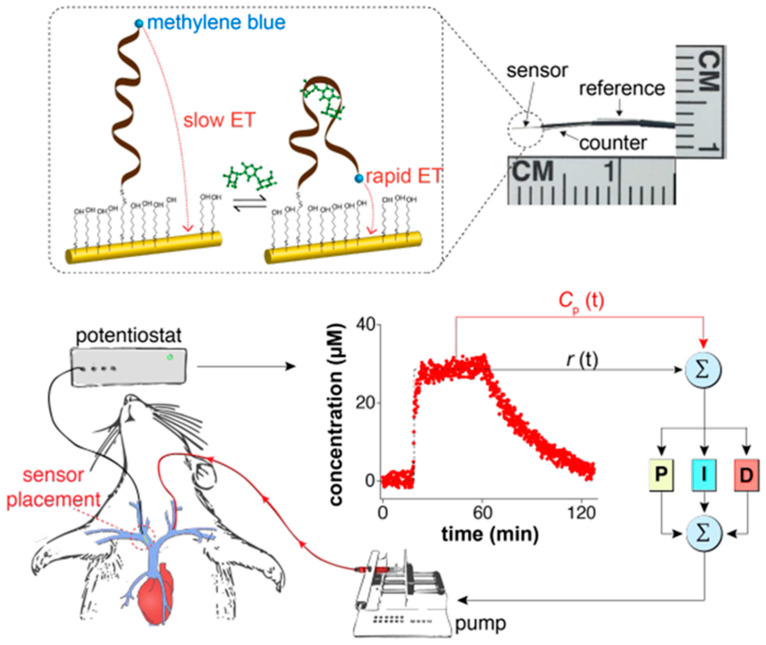
E-AB sensor implanted in the jugular of rats to monitor drug levels in vivo. Reprinted and adapted from [11] with permission.

Other interesting characteristics exhibited by this type of electrochemical biosensors include near real-time response, wash-free, reagentless, and single-step operation, reusability, and autonomous and selective enough read-outs. They were applied in multicomponent and protein-rich samples (blood serum, saliva, urine, seawater, soil suspensions, and foodstuffs such beer and milk). In addition, they have been operated in continuous mode in flowing undiluted samples (milk, blood, and secretions released by immune cells [26]) or even in situ within the living body (Figure 5) [2,3,10,11,12].

**Figure 6:**
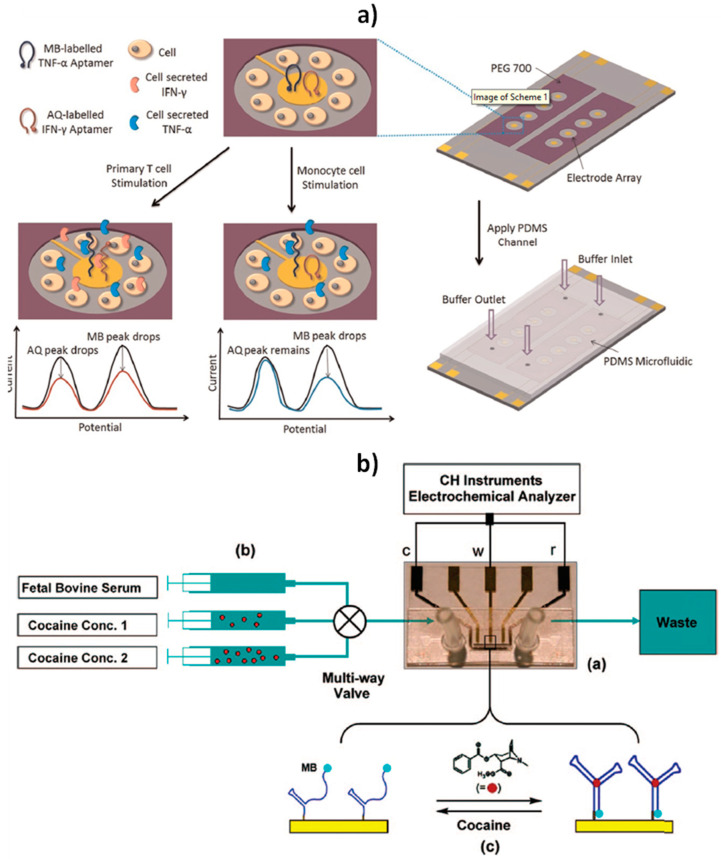
Schematic display of the microfluidic sensing platform using E-AB sensors for the dynamic monitoring of interferon-γ (IFN-γ) and tumor necrosis factor-α (TNF-α) from immune cells (**a**), and the experimental setup for the real time detection of cocaine in continuously flowing, undiluted blood serum using a E-AB sensor constructed onto a microfabricated MECAS chip (**b**). Reprinted and adapted from (a) [26] and (b) [9] with permission.

Other remarkable appealing characteristics and challenging achievements of these biosensors include their stability after storage for more than one week in room-temperature blood serum [6,61,71], their capability to respond to ups and downs in analyte concentration within seconds or minutes in a reversible way even in flowing complex samples without invoking reagents (which may contaminate the sample/product stream, or batch processing) [61,72], and their integration into microfluidic systems (Figure 6) [9,26].

It is important to mention that the great advances in nanotechnology and nanomaterials have allowed the development of other no-wash electrochemical biosensors apart from the folded-based ones [1,73,74]. However, they display some limitations that hamper their real-world application, such as signal drifting due to the change in environmental conditions, and electrode surface passivation and contamination after exposure to samples. 

Indeed, although they are little more than a decade old, the rational and relentless research on switch-based electrochemical biosensors, mainly by Plaxco’s group, has imparted these biosensors with additional attributes. As it is discussed in the following section, they have proved to be able to operate in flowing highly complex samples with the required accuracy and without the need for calibration.

### 2.3. Calibration-Free Biosensors

To achieve acceptable accuracy, electrochemical biosensors require calibration to correct for sensor-to-sensor fabrication variation and sensor drift. This requirement of calibration or recalibration several times a day (commercial continuous glucose monitors) has proven to be one of the significant hurdles limiting the widespread use of biosensors, decreasing convenience and increasing sensor complexity and cost and opportunities for errors, leading in turn to inappropriate clinical action [2,7]. The overcoming of this limitation is even more important and more complex for continuous, in vivo monitoring due to the drift invariably seen in sensors operating in situ within the body over many days. Under these challenging operation conditions, in-factory calibration has proven to be insufficient to assure clinical accuracy and reagent-using on-device autocalibration is impractical [7].

Calibration-free operating E-AB biosensors have been reported using two different ratiometric strategies: (1) the “dual-reporter” approach initially proposed by Ellington and coworkers [75] and (2) the “dual-frequency” operating method developed by Plaxco´s group [7]. Both strategies use as readout unit-less ratiometric values that are largely independent of sensor-to-sensor fabrication variation (attributed in this particular kind of biosensors to variations in electrode surface area and aptamer packing density) and sensor degradation. They overcome the irreproducibility of electrochemical DNA sensors and obviate the need for calibrating each individual sensor without scarifying sensitivity or selectivity. Importantly, both approaches are easily adaptable to nearly any electrochemical system that undergoes a change in its electron transfer kinetics in response to a target binding, and they may be employed in situ in the living body where calibrations are particularly difficult from a practical point of view [7]. 

**Figure 7:**
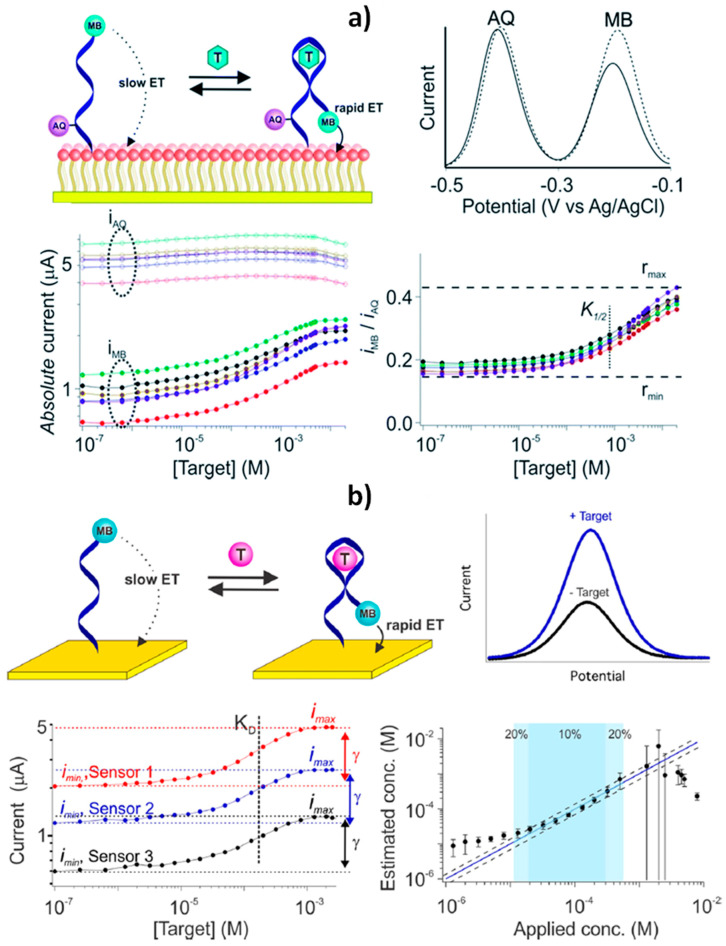
Schematic display of calibration-free E-AB biosensors based on “dual-reporter” (**a**) and “dual-frequency” (**b**) strategies. Reprinted and adapted from [2] (a) and [7] (b) with permission.

The “dual-reporter” strategy uses the ratio of the signal output provided by two different reporters (named as sensing and reference reporters), which are interrogated independently at non-overlapping redox potentials (Figure 7a) [2,75]. Conversely, the “dual-frequency” operating mode uses as output the ratio of peak currents collected at responsive and non- or low responsive square-wave frequencies (Figure 7b) [3,7].

Plaxco´s group demonstrated the potential of the “dual-frequency” interrogation strategy to develop calibration-free E-AB biosensors, exhibiting good accuracy and precision for the continuous measurement of two drugs in flowing whole blood over the course of hours, despite the significant drift observed in the absolute current recorded with the sensor [7]. The same group recently exploited this strategy to construct a calibration-free E-AB sensor for determining phenylalanine levels in blood compatible with POCT applications [3]. This biosensor can be deployed on screen-printed electrodes and allows the rapid (<10 min) determination of clinically relevant phenylalanine levels with an accuracy of ±20%, and specificity when challenged to in finger-prick-scale volumes of diluted unprocessed blood, thus offering the possibility to perform at-home measurements as an advance to personalized medicine.

Plaxco et al. also reported an E-AB biosensor combining the “dual reporter” approach [75] with drift-eliminating surface passivation using a phosphatidylcholine monolayer (Figure 7a) [12]. The biosensor was employed to perform calibration-free in vivo measurements of ATP or kanamycin using sensors placed in situ in the jugular veins of live rats over multi-hour measurements [2]. The “sensing” reporter (methylene blue, MB), was placed on the probe distal terminus and, therefore, the produced current was strongly modified by the conformational change associated with target recognition. The second, “reference,” reporter (anthraquinone, AQ) was placed in a position that responded differently to the presence of the target and served as an internal reference to correct the sensor-to-sensor variability. The use of a PC-terminated SAM (a phosphatidylcholine alkanethiolate derivative from 2-methacryloyloxyethyl phosphorylcholine) was largely responsible of the good biosensor functioning in vivo, allowing the elimination of the often severe-baseline drift observed in biosensors placed in the living body for long periods. It should be noted that the use of PC-terminated SAMs as backfilling agent demonstrated to be advantageous compared to the hydroxyl-terminated ones (the traditionally employed 6-mercapto-1-hexanol, MCH), lowering the baseline drift from around 70% to a 10% after 12 h in flowing whole blood in vitro or in situ in the veins of live animals. The method achieved precision in the micromolar range over many hours without invoking physical barriers (membranes or fluid sheaths to prevent cells from approaching the sensor surface, that increase the sensor bulk and slow sensor response times) or active drift-correction algorithms that require the collection of additional data at each time point, thereby degrading the time resolution.

## 3. Opportunities, Impact, Challenges, and Future Insights

With the aim to enhance the market adaptation and acceptance of electrochemical biosensors, significant progress has been made recently in the development of bioplatforms able to support continuous, real-time measurements of molecular biomarkers in unprocessed and/or flowing samples involving reagentless and single-step processes that are quantitative, easily multiplexed, and user-friendly for deployment at the POCT.

These advances have come hand in hand with rational modification of electrode surfaces with antibiofouling reagents (monolayers, transient polymeric coatings, or multifunctional peptides) or nanomaterials, and with relentless research into switch-based electrochemical biosensors and their coupling to ratiometric detection techniques.

According to the methods summarized in Table 1, one can deduce that there are electrochemical biosensors:

- Able to achieve high sensitivity and selectivity when defied punctually in multicomponent and protein-rich samples or continuously in flowing undiluted samples.
- Capable of responding to ups and downs in analyte concentration within seconds or minutes in a reversible way and without batch processing or addition of exogenous reagents.
- Insensitive to biofouling and stable after storage for more than one week in room-temperature blood serum.
- In ingestible formats coupled to transitory commercial polymer coatings with remarkable prolonged resistance to complex media of denaturing pH values such as gastrointestinal fluids.
- Integrated into microfluidic systems to monitor cell secretions.
- Deployed on screen-printed electrodes to provide rapid and accurate determinations when challenged to in finger-prick-scale sample volumes, suitable for application in POCT circumstances.
- Able to minimize the variability of the sensors fabrication and baseline drift and provide the required accuracy when operating continuously in vivo without the need for calibration, invoking physical barriers or using active drift-correction algorithms, thus surpassing main limitations of the commercial continuous glucose monitors.

We postulate these outstanding features beyond sensitivity and selectivity allow us to envision molecular detection moving away from complex, multi-step, benchtop assays towards direct, single-step devices (such as the home glucose monitor). These attributes will boost translational progress of electrochemical biosensors beyond the well-controlled laboratory benchtop into areas such as clinical diagnostics and field-portable devices and gain ground on other cumbersome methodologies to provide unprecedented quality control, safety monitoring, and clinical diagnosis even in resource limited areas. These impressive developments are decisive to deploy in vivo determinations, where the tuning of assay conditions is not so much inconvenient as it is impossible. 

These biosensors, competitive to gold standard molecular detection techniques as referred to clinical sensitivity and selectivity, are deemed to merge the quantitation and multiplexing of ELISAs with the convenience of use by nonspecialized user and speed of dipsticks, thus significantly improving current molecular detection. The detection limits achieved with ELISAs also come at a significant cost in terms of time, workflow, and equipment overhead that renders them not suitable for application at the POCT.

Compared to lateral-flow assays, which until recently have only given a binary output (“yes/no”) and request the user to read the test at an exact time, advanced electrochemical biosensors do not require a fixed readout time window, match them in terms of ease of use and surpass them in terms of the clinically relevant information they provide. Electrochemical bioplatforms provide quantitative and objective readout, thus giving a more precise picture of the tackled problem and allow the possibility to establish reliable clinical cut-off values. Moreover, unlike the dipsticks, which per design and per ease of use cannot integrate more than a few test lines, electrochemical biosensors are easily multiplexed to increase clinical sensitivity. However, there are many reasons why, so far, quantitative multiplexed biosensors are not popular and widespread in the market as lateral flow systems. Firstly, the interest in multiplexed determination to improve the reliability of the diagnostics is relatively recent. Secondly, multiplexing bioplatforms can be designed through two different approaches: using multielectrode arrays where each immunoreagent is attached to each electrode, or by means of barcode configurations involving a unique electrode platform and different electroactive labels with dissimilar electrochemical properties for each analyte. While multi-electrode arrays require complex and independent n-channel electrochemical workstations, the barcode approach makes use of distinct electroactive labels capable of generating appropriate and distinguishable signals at different potentials in a single amperometric or voltammetric scan. Unfortunately, the number of different electroactive labels with dissimilar electrochemical properties is quite limited. Therefore, the development of low-cost, custom designed, and field-portable multiplexed potentiostats and the use of novel nanomaterials and/or electroactive probes producing independent electrochemical signals is required. In addition, multiplexed quantitative bioplatforms should be adopted by the individual user in a clinical environment. This, in turn, requires both the identification and clinical validation of new and reliable signatures of biomarkers for each particular application and what is more, laboratory personnel gains familiarity with the new methodologies, and medical personnel themselves expand their knowledge to implement the technology and produce trustworthy results interpretation. Moreover, since the identified biomarkers panel could comprise biomarkers with high differences in the threshold levels, additional efforts should be focused on developing electrochemical biosensing strategies suitable to simultaneously determine biomarkers at very different concentration levels and both of genetic or protein nature.

Despite the great strides made, other research efforts should also be devoted to couple electrochemical biosensing devices with nanozymes and implementation on paper-based substrates. These advances would impart unprecedented opportunities upon electrochemical biosensors in terms of cost, stability, and eco-friendliness, and would allow us to face them with a large number of samples, multi-determination of several analytes, and continuous quantitative analysis for long times, by different users and in different environments beyond the well-controlled laboratory benchtop. Furthermore, although advanced sensors are envisioned to be a part of many more emerging technologies such as wearable devices, significant development stemming from multidisciplinary efforts in material sciences, electrochemistry, biophysics, biology, and pharmacology will be needed. Funding of innovative R&D and productive collaboration between universities, research centers, companies, and end users should also be enhanced to face up to the continuous evolving market demands. 

Other attributes worth pursuing include tuning the concentration range (matching the specificity window of the receptor with the expected range of target concentrations in a given application) and the selectivity (minimizing cross-reactivity with close structural analogues of the targeted molecule) of biomolecular receptors through the rational adaptation of two strategies employed by nature, structure-switching and allosteric control, and expanding the variety of analytes to be detected by exploring other type of receptors in switch-based electrochemical biosensors.

In summary, although there are still several bottlenecks to overcome and we must be aware that there are no yet many commercially available electrochemical biosensors, the intense work performed and in due course to push the outstanding and unique opportunities they provide brings us closer and closer to this point. This will aid with having devices designed on demand, which will end up offering any attribute we can imagine while requiring less and less attention to do their job. The great promise they offer to largely simplify and speed up molecular detection makes electrochemical biosensors particularly appealing to support a broad range of applications while considerably improving our quality and way of life. 

We hope this review will help newcomers to the field catch up with the current state of the art technology and will stimulate new researchers to join those with long experience in this field to continue to work on bringing out their full potential.

## Figures and Tables

**Table 1:** Representative electrochemical (bio)sensors exhibiting remarkable sensing attributes beyond sensitivity and selectivity.

Electrode	Sensor Fundamentals	Transduction Technique	Attribute (used Approach)	Additional Features	Molecular Target Tested	L.R./LOD	Sample	Ref.
16× Au electrode arrays prepared by photolithography	Sandwich hybridization assay at arrays modified with SHCP/HDT+MCH	Chrono-amperometry (TMB/H_2_O_2_)	Antibiofouling (thiolated ternary monolayer)	—	Synthetic target DNA (characteristic region of *E. coli* 16S rRNA)	LOD: 7 pM and 17 pM in spiked undiluted human serum and urine	Raw undiluted human serum and urine	[20]
Au/SPEs	Sandwich hybridization assay at arrays modified with SHCP/HDT+MCH	Chrono-amperometry (TMB/H_2_O_2_)	Antibiofouling (thiolated ternary monolayer)	—	Synthetic target DNA and *E. coli* 16S rRNA	LOD: 25 pM and 100 pM in spiked undiluted human serum and urine and 16S rRNA *E. coli* corresponding to 3000 CFU mL^−^^1^ in raw cell lysate samples	Untreated raw serum, urine, and crude bacterial lysate solutions	[21]
AuE	E-AB: Aptamer dually modified with a thiol and a redox reporter + PC-terminated SAM	SWV (MB)	Antibiofouling (PC-terminated SAM)	Continuous operation label-free	Kanamycin, doxorubicin	—	Flowing whole blood, both in vitro and in vivo (sensors placed in the jugular veins of live rats)	[12]
GOx-PB-graphite SPEs	Electrode modified with Eudragit^®^ L100	CV ([Fe(CN)_6_]^4−/3−^) and Chrono-amperometry (PB/H_2_O_2_)	Antibiofouling (pH-sensitive transient polymer coating)	Continuous operation	Glucose	—	Raw undiluted blood and saliva	[22]
Edible carbon paste GOx biosensors	Electrodes coated with Eudragit^®^ E PO (pH < 5.0) or Eudragit® L100 (pH > 6.0)	Chrono-amperometry (PB/H_2_O_2_)	Antibiofouling (pH-sensitive transient polymer coating)	Continuous operation Biocatalytic activity preservation at media with denaturing pH values	Glucose	L.R.: 2−10 mM	GI fluids	[23]
PEDOT-citrate film-modified GCE	Covalent immobilization using EDC/NHS chemistry of a peptide with anchoring, antifouling, and recognizing capabilities onto GCE/PEDOT-citrate	DPV ([Fe(CN)_6_]^4−/3−^)	Antibiofouling (multifunctional peptide)	—	APN, HepG2 cells	L.R.: 1 ng mL^−1^−15 μg mL^−1^ (APN) and 50–5 × 10^5^ cells mL^−1^ (HepG2 cells) LOD: 0.4 ng mL^−1^ (APN) and 20 cells mL^−1^ (HepG2 cells)	Human urine	[24]
Au disk	E-DNA: DNA probe dually modified with a thiol and a redox reporter + MCH SAM	SWV (MB)	Continuous and real-time operation (Folded-biosensor)	Reagentless and single-step	Melamine	LOD: 150 μM (∼19 ppm) in buffered solutions and 20 μM (∼2.5 ppm) in whole milk	Flowing undiluted whole milk	[6]
Au	E-DNA: TDNs with two functional DNA/aptamer strands, one of them modified with MB	SWV (MB)	Continuous and real-time operation (Folded-biosensor)	Reagentless and single-step Antibiofouling Reusability	Target DNA, ATP	LOD: 300 fM (target DNA), 5 nM (ATP)	Flowing whole blood	[8]
AuE	E-DNA: nucleic acid “scaffold” attached on one end to an electrode and presenting both a redox reporter and a specific epitope on the other	SWV (MB)	Reagentless and single-step (Folded-biosensor)	—	Three types of HIV-diagnostic antibodies	—	Human serum	[25]
Microfabricated Au onto MECAS chip	E-AB: Aptamer dually modified with a thiol and a redox reporter + MCH SAM	ACV (MB)	Continuous and real-time operation (Folded-biosensor)	Reagentless and single-step Reusability	Cocaine	—	Flowing undiluted blood serum	[9]
100 nm Au layer sputtered on glass slides	E-AB: Hairpin structure aptamer dually modified with a thiol and a redox reporter (MB or AQ) + MCH SAM	SWV (MB, AQ)	Continuous operation (Folded-biosensor)	Antibiofouling Reagentless and single-step	IFN-γ + TNF-α	LOD: 6.35 ng mL^−1^ (IFN-γ), 5.46 ng mL^−1^ (TNF-α)	Integrated into microfluidic devices to dynamically monitoring of cytokine release from immune cells (2.5 h)	[26]
Au wire	E-AB: Aptamer dually modified with a thiol and a redox reporter + MCH SAM	SWV (MB)	Continuous and real-time and in vivo operation (Folded-biosensor)	Reagentless and single-step	Doxorubicin, Kanamycin, Gentamycin, and Tobramycin	—	Bloodstream awake, ambulatory rats	[10]
Au disk, Au-plated SPCEs	E-PB: Peptide dually modified with a thiol and a redox reporter + MCH SAM	ACV, CV (MB)	Real-time operation (Folded-biosensor)	Reagentless and single-step	Pb^2+^	LOD: 5 μM (ACV)	Diluted tap water, saliva, and urine samples	[27]
Au disk	E-ION: T-rich ssDNA dually modified with thiol and redox reporter + Hg^2+^ + MCH SAM	ACV (MB)	Real-time operation (Folded-biosensor)	Reagentless and single-step Reusable	GSH (displaces Hg^2+^ by chelation)	LOD: 5 nM	50% synthetic human saliva	[28]
AuE	E-AB: Aptamer dually modified with a thiol and a redox reporter + MCH SAM	SWV (MB)	Calibration-free (“dual-frequency”)	Continuous and real-time operation Reagentless and single-step	Cocaine, doxorubicin	—	Continuous measurement in flowing, undiluted whole blood	[7]
Au-SPE	E-AB: Aptamer dually modified with a thiol and a redox reporter + MCH	SWV (MB)	Calibration-free (“dual-frequency”)	Reagentless and single-step	Phenylalanine	L.R.: 90 nM−7 μM	Whole blood (diluted 1000-fold to match the sensor’s dynamic range)	[3]
AuE	E-AB: Aptamer modified with a thiol and two different redox reporters + PC-terminated SAM	SWV (MB and AQ)	Calibration-free, (“dual reporter”) and in vivo operation	Continuous operation Antibiofouling Reagentless and single-step	Cocaine, ATP, kanamycin	—	Flowing whole blood, both in vitro and in vivo (sensors placed in the jugular veins of live rats)	[2]

Abbreviations: ACV: alternating current voltammetry; APN: aminopeptidase N; AQ: anthraquinone; ATP: anti-adenosine triphosphate; CFU: colony forming unit; CV: cyclic voltammetry; DPV: differential pulse voltammetry; E-AB: electrochemical aptamer-based; *E. coli*: *Escherichia coli*; EDC: carbodiimide; E-DNA: electrochemical DNA-based biosensor; E-ION: electrochemical for ion determination; E-PB: electrochemical peptide-based biosensor; GCE: glassy carbon electrode; GI: gastrointestinal fluids; GOx: glucose oxidase; GSH: glutathione; HDT: 1,6-hexanedithiol; HIV: Human Immunodeficiency Virus; INF-γ: interferon-γ; LOD: limit of detection; L.R.: linear range; MB: methylene blue; MCH: 6-mercapto-1-hexanol; MECAS: Microfluidic Electrochemical Aptamer-based Sensor; NHS: succinimide; PB: Prussian Blue; PC: phosphatidylcholine; PEDOT: poly (3,4-ethylenedioxythiophene); SAM: self-assembled monolayer; SHCP: thiolated capture probe; SPCEs: screen-printed carbon electrodes; SPEs: screen-printed electrodes; SWV: square wave voltammetry; T: thymine; TDNs: tetrahedral DNA nanostructures; TMB: tetramethylbenzidine; TNF-α: tumor necrosis factor-α.

## References

[1] Zhang Y., Chen X. (2019). Nanotechnology and nanomaterials-based no-wash electrochemical biosensors: From design to application. Nanoscale.

[2] Li H., Li S., Dai J., Li C., Zhu M., Li H., Lou X., Xia F., Plaxco K.W. (2019). High frequency, calibration-free molecular measurements in situ in the living body. Chem. Sci..

[3] Idili A., Parolo C., Ortega G., Plaxco K.W. (2019). Calibration-free measurement of phenylalanine levels in the blood using an electrochemical aptamer-based sensor suitable for point-of-care applications. ACS Sens..

[4] Duan D., Fan K., Zhang D., Tan S., Liang M., Liu Y., Zhang J., Zhang P., Liu W., Qiu X. (2015). Nanozyme-strip for rapid local diagnosis of Ebola. Biosens. Bioelectron..

[5] Qiu W., Baryeh K., Takalkar S., Chen W., Liu G. (2019). Carbon nanotube-based lateral flow immunoassay for ultrasensitive detection of proteins: Application to the determination of IgG. Microchim. Acta.

[6] Li H., Somerson J., Xia F., Plaxco K.W. (2018). Electrochemical DNA-based sensors for molecular quality control: Continuous, real-time melamine detection in flowing whole milk. Anal. Chem..

[7] Li H., Dauphin-Ducharme P., Ortega G., Plaxco K.W. (2017). Calibration-free electrochemical biosensors supporting accurate molecular measurements directly in undiluted whole blood. J. Am. Chem. Soc..

[8] Li C., Hu X., Lu J., Mao X., Xiang Y., Shu Y., Li G. (2018). Design of DNA nanostructure-based interfacial probes for the electrochemical detection of nucleic acids directly in whole blood. Chem. Sci..

[9] Swensen J.S., Xiao Y., Ferguson B.S., Lubin A.A., Lai R.Y., Heeger A.J., Plaxco K.W., Soh H.T. (2009). Continuous, real-time monitoring of cocaine in undiluted blood serum via a microfluidic, electrochemical aptamer-based sensor. J. Am. Chem. Soc..

[10] Arroyo-Currás N., Somerson J., Vieira P.A., Ploense K.L., Kippine T.E., Plaxco K.W. (2017). Real-time measurement of small molecules directly in awake, ambulatory animals. Proc. Natl. Acad. Sci. USA.

[11] Arroyo-Currás N., Ortega G., Copp D.A., Ploense K.L., Plaxco Z.A., Kippin T.E., Hespanha J.P., Plaxco K.W. (2018). High-precision control of plasma drug levels using feedback-controlled dosing. ACS Pharmacol. Transl. Sci..

[12] Li H., Dauphin-Ducharme P., Arroyo-Currás N., Tran C.H., Vieira P.A., Li S., Shin C., Somerson J., Kippin T.E., Plaxco K.W. (2017). A biomimetic phosphatidylcholine-terminated monolayer greatly improves the in vivo performance of electrochemical aptamer-based sensors. Angew. Chem. Int. Ed..

[13] Bissonnette S., del Grosso E., Simon A.J., Plaxco K.W., Ricci F., Vallée-Bélisle A. (2020). Optimizing the specificity window of biomolecular receptors using structure-switching and allostery. ACS Sens..

[14] Kim J., Kumar R., Bandodkar A.J., Wang J. (2017). Advanced materials for printed wearable electrochemical devices: A review. Adv. Electron. Mater..

[15] Hubble L.J., Wang J. (2019). Sensing at Your Fingertips: Glove-based wearable chemical sensors. Electroanalysis.

[16] Khan S., Ali S., Bermak A. (2019). Recent developments in printing flexible and wearable sensing electronics for healthcare applications. Sensors.

[17] Tu J., Torrente-Rodríguez R.M., Wang M., Gao W. (2019). The era of digital health: A review of portable and wearable affinity biosensors. Adv. Funct. Mater..

[18] Kim J., Campbell A.S., Esteban-Fernández de Ávila B., Wang J. (2019). Wearable biosensors for healthcare monitoring. Nat. Biotechnol..

[19] Sempionatto J.R., Jeerapan I., Krishnan S., Wang J. (2020). Wearable chemical sensors: Emerging systems for on-body analytical chemistry. Anal. Chem..

[20] Campuzano S., Kuralay F., Lobo-Castañón M.J., Bartošik M., Vyavahare K., Paleček E., Haake D.A., Wang J. (2011). Ternary monolayers as DNA recognition interfaces for direct and sensitive electrochemical detection in untreated clinical samples. Biosens. Bioelectron..

[21] Kuralay F., Campuzano S., Haake D.A., Wang J. (2011). Highly sensitive disposable nucleic acid biosensors for direct bioelectronic detection in raw biological samples. Talanta.

[22] Ruiz-Valdepeñas Montiel V., Sempionatto J.R., Esteban-Fernández de Ávila B., Whitworth A., Campuzano S., Pingarrón J.M., Wang J. (2018). Delayed sensor activation based on transient coatings: Biofouling protection in complex biofluids. J. Am. Chem. Soc..

[23] Ruiz-Valdepeñas Montiel V., Sempionatto J.R., Campuzano S., Pingarrón J.M., Esteban-Fernández de Ávila B., Wang J. (2019). Direct electrochemical biosensing in gastrointestinal fluids. Anal. Bioanal. Chem..

[24] Song Z., Chen M., Ding C., Luo X. (2020). Designed three-in-one peptides with anchoring, antifouling and recognizing capabilities for highly sensitive and low fouling electrochemical sensing in complex biological media. Anal. Chem..

[25] Parolo C., Greenwood A.S., Ogden N.E., Kang D., Hawes C., Ortega G., Arroyo-Currás N., Plaxco K.W. (2020). E-DNA scaffold sensors and the reagentless, single step, measurement of HIV-diagnostic antibodies in human serum. Microsyst. Nanoeng..

[26] Liu Y., Liu Y., Matharu Z., Rahimian A., Revzin A. (2015). Detecting multiple cell-secreted cytokines from the same aptamer functionalized electrode. Biosens. Bioelectron..

[27] Zhad H.R.L.Z., Lai R.Y. (2018). Application of calcium-binding motif of E-cadherin for electrochemical detection of Pb(II). Anal. Chem..

[28] Zhad H.R.L.Z., Lai R.Y. (2014). A Hg(II)-mediated ‘‘signal-on’’ electrochemical glutathione sensor. Chem. Commun..

[29] Campuzano S., Pedrero M., Yáñez-Sedeño P., Pingarrón J.M. (2019). Antifouling (bio)materials for electrochemical (bio)sensing. Int. J. Mol. Sci..

[30] Cui M., Wang Y., Jiao M., Jayachandran S., Wu Y., Fan X., Luo X. (2017). Mixed self-assembled aptamer and newly designed zwitterionic peptide as antifouling biosensing interface for electrochemical detection of alpha-fetoprotein. ACS Sens..

[31] Wang G., Han R., Sua X., Lia Y., Xua G., Luo X. (2017). Zwitterionic peptide anchored to conducting polymer PEDOT for the development of antifouling and ultrasensitive electrochemical DNA sensor. Biosens. Bioelectron..

[32] Wang Y., Cui M., Jiao M., Luo X. (2018). Antifouling and ultrasensitive biosensing interface based on self-assembled peptide and aptamer on macroporous gold for electrochemical detection of immunoglobulin E in serum. Anal. Bioanal. Chem..

[33] Henry O.Y.F., Acero Sanchez J.L., O’Sullivan C.K. (2010). Bipodal PEGylated alkanethiol for the enhanced electrochemical detection of genetic markers involved in breast cancer. Biosens. Bioelectron..

[34] Dharuman V., Chang B.-Y., Park S.-M., Hahn J.H. (2010). Ternary mixed monolayers for simultaneous DNA orientation control and surface passivation for label free DNA hybridization electrochemical sensing. Biosens. Bioelectron..

[35] Dharuman V., Vijayaraj K., Radhakrishnan S., Dinakaran T., Narayanan J.S., Bhuvana M., Wilson J. (2011). Sensitive label-free electrochemical DNA hybridization detection in the presence of 11-mercaptoundecanoic acid on the thiolated single strand DNA and mercaptohexanol binary mixed monolayer surface. Electrochim. Acta.

[36] Campuzano S., Kuralay F., Wang J. (2012). Ternary monolayer interfaces for ultrasensitive and direct bioelectronics detection of nucleic acids in complex matrices. Electroanalysis.

[37] Blaszykowski C., Sheikh S., Thompson M. (2012). Surface chemistry to minimize fouling from blood-based fluids. Chem. Soc. Rev..

[38] Josephs E.A., Ye T. (2012). A Single-Molecule View of conformational switching of DNA tethered to a gold electrode. J. Am. Chem. Soc..

[39] Halford C., Gonzalez R., Campuzano S., Hu B., Babbitt J.T., Liu J., Wang J., Churchill B.M., Haake D.A. (2013). Rapid antimicrobial susceptibility testing by sensitive detection of precursor rRNA using a novel electrochemical biosensing platform. Antimicrob. Agents Chemother..

[40] Goda T., Tabata M., Sanjoh M., Uchimura M., Iwasaki Y., Miyahara Y. (2013). Thiolated 2-methacryloyloxyethyl phosphorylcholine for an antifouling biosensor platform. Chem. Commun..

[41] McQuistan A., Zaitouna A.J., Echeverria E., Lai R.Y. (2014). Use of thiolated oligonucleotides as anti-fouling diluents in electrochemical peptide-based sensors. Chem. Commun..

[42] Jolly P., Formisano N., Tkáč J., Kasák P., Frost C.G., Estrela P. (2015). Label-free impedimetric aptasensor with antifouling surface chemistry: A prostate specific antigen case study. Sens. Actuators B Chem..

[43] González-Fernández E., Avlonitis N., Murray A.F., Mount A.R., Bradley M. (2016). Methylene blue not ferrocene: Optimal reporters for electrochemical detection of protease activity. Biosens. Bioelectron..

[44] Hu Y., Liang B., Fang L., Ma G., Yang G., Zhu Q., Chen S., Ye X. (2016). Antifouling Zwitterionic Coating via Electrochemically Mediated Atom Transfer Radical Polymerization on Enzyme-Based Glucose Sensors for Long-Time Stability in 37 °C Serum. Langmuir.

[45] Gao J., Jeffries L., Mach K.E., Craft D.W., Thomas N.J., Gau V., Liao J.C., Wong P.K. (2017). A multiplex electrochemical biosensor for bloodstream infection diagnosis. SLAS Technol..

[46] Altobelli E., Mohan R., Mach K.E., Sin M.L.Y., Anikst V., Buscarini M., Wong P.K., Gau V., Banaei N., Liao J.C. (2017). Integrated biosensor assay for rapid uropathogen identification and phenotypic antimicrobial susceptibility testing. Eur. Urol. Focus.

[47] Ding S., Mosher C., Lee X.Y., Das S.R., Cargill A.A., Tang X., Chen B., McLamore E.S., Gomes C., Hostetter J.M. (2017). Rapid and label-free detection of interferon gamma via an electrochemical aptasensor comprising a ternary surface monolayer on a gold interdigitated electrode array. ACS Sens..

[48] Miranda-Castro R., Sánchez-Salcedo R., Suárez-Álvarez B., de-los-Santos-Álvarez N., Miranda-Ordieres A.J., Lobo-Castañón M.J. (2017). Thioaromatic DNA monolayers for target-amplification-free electrochemical sensing of environmental pathogenic bacteria. Biosens. Bioelectron..

[49] Miranda-Castro R., de-los-Santos-Álvarez N., Lobo-Castañón M.J. (2018). Understanding the factors affecting the analytical performance of sandwich-hybridization genosensors on gold electrodes. Electroanalysis.

[50] González-Fernández E., Staderini M., Yussof A., Scholefield E., Murray A.F., Mount A.R., Bradley M. (2018). Electrochemical sensing of human neutrophil elastase and polymorphonuclear neutrophil activity. Biosens. Bioelectron..

[51] González-Fernández E., Staderini M., Avlonitis N., Murray A.F., Mount A.R., Bradley M. (2018). Effect of spacer length on the performance of peptide-based electrochemical biosensors for protease detection. Sens. Actuators B Chem..

[52] Boozer C., Chen S.F., Jiang S.Y. (2006). Controlling DNA orientation on mixed ssDNA/OEG SAMs. Langmuir.

[53] Dharuman V., Hahn J.H. (2008). Label-free electrochemical DNA hybridization discrimination effects at the binary and ternary mixed monolayers of single stranded DNA/diluent/s in presence of cationic intercalators. Biosens. Bioelectron..

[54] Wu J., Campuzano S., Halford C., Haake D.A., Wang J. (2010). Ternary surface monolayers for ultrasensitive (zeptomole) amperometric detection of nucleic acid hybridization without signal amplification. Anal. Chem..

[55] Goodman R.P., Berry R.M., Turberfield A.J. (2004). The single-step synthesis of a DNA tetrahedron. Chem. Commun..

[56] Pei H., Lu N., Wen Y., Song S., Liu Y., Yan H., Fan C. (2010). A DNA Nanostructure-based biomolecular probe carrier platform for electrochemical biosensing. Adv. Mater..

[57] Lin M., Song P., Zhou G., Zuo X., Aldalbahi A., Lou X., Shi J., Fan C. (2016). Electrochemical detection of nucleic acids, proteins, small molecules and cells using a DNA-nanostructure-based universal biosensing platform. Nat. Protoc..

[58] Dong S., Zhao R., Zhu J., Lu X., Li Y., Qiu S., Ji L., Jiao X., Song S., Fan C. (2015). Electrochemical DNA biosensor based on a tetrahedral nanostructure probe for the detection of Avian Influenza A (H7N9) virus. ACS Appl. Mater. Interfaces.

[59] Lubin A.A., Plaxco K.W. (2010). Folding-based electrochemical biosensors: The case for responsive nucleic acid architectures. Acc. Chem. Res..

[60] Campuzano S., Yáñez-Sedeño P., Pingarrón J.M. (2019). Reagentless and reusable electrochemical affinity biosensors for near real-time and/or continuous operation. Advances and prospects. Curr. Opin. Electrochem..

[61] Plaxco K.W., Soh H.T. (2011). Switch-based biosensors: A new approach towards real-time, in vivo molecular detection. Trends Biotechnol..

[62] Arroyo-Currás N., Dauphin-Ducharme P., Scida K., Chávez J.L. (2020). From the beaker to the body: Translational challenges for electrochemical, aptamer-based sensors. Anal. Methods.

[63] Ranallo S., Rossetti M., Plaxco K.W., Vallée-Bélisle A., Ricci F. (2015). A modular, DNA-based beacon for single-step fluorescence detection of antibodies and other proteins. Angew. Chem. Int. Ed..

[64] White R.J., Kallewaard H.M., Hsieh W., Patterson A.S., Kasehagen J.B., Cash K.J., Uzawa T., Soh H.T., Plaxco K.W. (2012). Wash-free, electrochemical platform for the quantitative, multiplexed detection of specific antibodies. Anal. Chem..

[65] Ferguson B.S., Hoggarth D.A., Maliniak D., Ploense K., White R.J., Woodward N., Hsieh K., Bonham A.J., Eisenstein M., Kippin T.E. (2013). Real-time, aptamer-based tracking of circulating therapeutic agents in living animals. Sci. Transl. Med..

[66] Kang D., Sun S., Kurnik M., Morales D., Dahlquist F.W., Plaxco K.W. (2017). New architecture for reagentless, protein-based electrochemical biosensors. J. Am. Chem. Soc..

[67] Xiao Y., Lubin A.A., Baker B.R., Plaxco K.W., Heeger A.J. (2006). Single-step electronic detection of femtomolar DNA by target-induced strand displacement in an electrode-bound duplex. Proc. Natl. Acad. Sci. USA.

[68] Wang Q., Gao F., Ni J., Liao X., Zhang X., Lin Z. (2016). Facile construction of a highly sensitive DNA biosensor by in-situ assembly of electro-active tags on hairpin-structured probe fragment. Sci. Rep..

[69] Zaitouna A.J., Lai R.Y. (2014). An electrochemical peptide-based Ara h 2 antibody sensor fabricated on a nickel(II)-nitriloacetic acid self-assembled monolayer using a His-tagged peptide. Anal. Chim. Acta.

[70] Mayer M.D., Lai R.Y. (2018). Effects of redox label location on the performance of an electrochemical aptamer-based tumor necrosis factor-alpha sensor. Talanta.

[71] Lai R.Y., Seferos D.S., Heeger A.J., Bazan G.C., Plaxco K.W. (2016). Comparison of the signaling and stability of electrochemical DNA sensors fabricated from 6- or 11-carbon self-assembled monolayers. Langmuir.

[72] Baker B.R., Lai R.Y., Wood M.S., Doctor E.H., Heeger A.J., Plaxco K.W. (2006). An electronic, aptamer-based small-molecule sensor for the rapid, label-free detection of cocaine in adulterated samples and biological Fluids. J. Am. Chem. Soc..

[73] Parate K., Karunakaran C., Claussen J.C. (2019). Electrochemical cotinine sensing with a molecularly imprinted polymer on a graphene-platinum nanoparticle modified carbon electrode towards cigarette smoke exposure monitoring. Sens. Actuators B Chem..

[74] Wang Z., Ma B., Shen C., Cheong L.Z. (2019). Direct, selective and ultrasensitive electrochemical biosensing of methyl parathion in vegetables using Burkholderia cepacia lipase@MOF nanofibersbased biosensor. Talanta.

[75] Du Y., Lim B.J., Li B., Jiang Y.S., Sessler J.L., Ellington A.D. (2014). Reagentless, ratiometric electrochemical DNA sensors with improved robustness and reproducibility. Anal. Chem..

